# FOXM1 and polo-like kinase 1 are co-ordinately overexpressed in patients with gastric adenocarcinomas

**DOI:** 10.1186/s13104-015-1658-y

**Published:** 2015-11-14

**Authors:** M. Dibb, N. Han, J. Choudhury, S. Hayes, H. Valentine, C. West, AD Sharrocks, Yeng S. Ang

**Affiliations:** Faculty of Life Sciences, University of Manchester, Michael Smith Building, Oxford Road, Manchester, M13 9PT UK; Faculty of Medical and Human Sciences, University of Manchester, Oxford Road, Manchester, UK; Department of Histopathology, Salford Royal Foundation Trust, Stott Lane, Salford, M6 8HD UK; School of Cancer and Enabling Sciences, Christie Hospital, Manchester Academic Health Science Centre, The University of Manchester, Manchester, UK; GI Science Centre, Salford Royal NHS FT, University of Manchester, Stott Lane, Salford, M6 8HD UK

**Keywords:** Gastric adenocarcinoma, FOXM1 (Forkhead Protein M1), PLK1 (Polo-like Kinase 1)

## Abstract

**Background:**

Gastric cancers present late in life with advanced disease and carry a poor prognosis. Polo-like Kinase 1 (PLK1) is a mitotic kinase with regulatory functions during G2/M and mitosis in the cell cycle. In mammalian cells, there is an intricate co-regulatory relationship between PLK1 and the forkhead transcription factor FOXM1. It has been demonstrated that individually either PLK1 or FOXM1 expression predicts poorer survival. However, the co-expression of both of these markers in gastric adenocarcinomas has not been reported previously.

**Methods:**

We aimed to assess the expression of PLK1 and FOXM1 in Gastric adenocarcinomas in a Western Population, to examine whether there is a relationship of PLK1 to FOXM1 in cancer samples. We assess both the protein and mRNA expression in this patient population by Tissue Microarray immunohistochemistry and RT-PCR.

**Results:**

Immunohistochemistry was performed on biopsy samples from 79 patients with gastric cancer. Paired normal controls were available in 47 patients. FOXM1 expression was significantly associated with gastric adenocarcinoma (p = 0.001). *PLK1* and *FOXM1* co-expression was demonstrated in 6/8 (75 %) tumours when analysed by RT-PCR. FOXM1 is overexpressed in a large proportion of gastric carcinomas at the protein level and *FOXM1* and *PLK1* are concomitantly overexpressed at the mRNA level in this cancer type.

**Conclusions:**

This study has demonstrated that FOXM1 and its target gene *PLK1* are coordinately overexpressed in a proportion of gastric adenocarcinomas. This suggests that chemotherapeutic treatments that target this pathway may be of clinical utility.

## Background

Gastric adenocarcinoma remains the second commonest cause of cancer death on a worldwide basis. In the West, the incidence has been steadily declining over the past few decades [1, 2]. However, in the United Kingdom gastric cancer remains the sixth commonest cause of cancer death [3]. This is mainly due to late presentation of the disease and thus limits treatment options. The 5-year survival rate is good if the disease is diagnosed at an early stage and large scale population screening by upper gastrointestinal endoscopy has increased the rates of early detection and subsequent prognosis in Japanese populations [4, 5]. However, the incidence of gastric adenocarcinoma is much lower in the West and large scale population screening is not cost effective. Consequently, gastric adenocarcinomas are usually diagnosed at an advanced stage and typical 5-year survival is less than 15 %. Traditional cytotoxic chemotherapy regimens are largely ineffective in halting the disease [6–8]. Even when surgery is indicated, neoadjuvant chemotherapy improves 5-year survival rates modestly from 20 to 36 % [9]. Hence novel treatments and targets for drug therapies are urgently needed to improve outcomes further.

FOXM1 is a member of the forkhead transcription factor family, which plays an important role in controlling the cell cycle [10, 11]. Specifically, FOXM1 controls mitotic entry through the periodic upregulation of a group of genes that are maximally expressed as cells progress through late G2 and into M phase [12]. Two of its target genes are *CCNB1* and *PLK1*, and these form part of a kinase-driven positive feedback loop that leads to the phosphorylation of FOXM1 and potentiation of its activity [13, 14]. Hence, there is an intricate inter-regulatory relationship between FOXM1 and PLK1 that creates a cellcycle control switch. This important link between FOXM1 and cellcycle control suggests that it is likely to contribute to the aberrant cell proliferation associated with malignancy. In clinical settings, *FOXM1* has been shown to be upregulated in a range of different tumour types [11]. More recently, FOXM1 was shown to be overexpressed and of potential prognostic significance in oesophageal adenocarcinomas and squamous carcinomas [15, 16]. In the same fashion, the FOXM1 target gene *PLK1* has also been shown to be overexpressed in a wide range of tumours [17], including oesophageal adeno- and squamous carcinomas [15, 18, 19]. Furthermore, novel molecular functions for FOXM1 have been identified in cancer cells beyond simply the acceleration of G2–M phase progression [11, 20]. This is exemplified by its ability to promote the nuclear translocation of *β*-catenin in gliomas, and consequently it can activate a whole programme of Wnt target genes [21]. Taken together, these findings indicate that FOXM1 and PLK1 are likely central regulators in carcinogenesis and are therefore potential therapeutic targets.

In the current study, we investigate the expression of FOXM1 and PLK1 in gastric adenocarcinomas with particular emphasis on examining whether there is evidence that FOXM1and PLK1 are co-ordinately expressed. We aimed to show that there is a relationship of PLK1 to FOXM1 in cancer samples. Such co-regulation in cancer samples has not previously been investigated; as studies have been limited to studying either FOXM1 or PLK1 expression in these tumours [22, 23]. We previously demonstrated co-upregulation of these proteins in oesophageal adenocarcinomas [15]. We now show that there is also coordinate overexpression of *FOXM1* and *PLK1* in gastric adenocarcinomas, thereby providing the potential for feedback potentiation of FOXM1 activity. PLK1 inhibitors are currently being developed for cancer therapy [17, 24] and it is likely that cells demonstrating coordinated up regulation of FOXM1 and PLK1 expression will be particularly susceptible to such treatment.

## Methods

### Tissue collection

The Wrightington, Wigan and Leigh NHS Local Research and Ethics Committee initially granted ethical approval in 2007. Tissue was obtained a total of 79 gastric carcinomas and 47 healthy controls have been directly recruited. Patients who had received neo-adjuvant chemotherapy were excluded. Paraffin Blocks were also used to construct slides for immunohistochemistry.

Demographic Information, including age at diagnosis, sex, co-morbidity and survival was collected on all patients. Tumour grade and stage were documented using the TNM and AJCC criteria. Biopsy samples were collected during endoscopy from the tumour and where possible from macroscopically normal adjacent tissue at least 5 cm away from macroscopic evidence of tumour. Endoscopic biopsy samples were then either snap-frozen in liquid nitrogen or collected in RNAlater (Qiagen Laboratories)©. They were then archived at −80 °C until needed. A small number of surgical samples were also taken. These were dissected from the tumour and adjacent normal tissue by a histopathologist immediately after removal from the patient. Surgical samples were frozen in liquid nitrogen and then stored at −80 °C. Samples were used for RNA extraction and protein analysis.

### Immunohistochemistry

Tissue microarray (TMA) blocks were constructed from surgical tumour block resections and biopsies as described previously in our laboratory [15]. Three arrays were constructed for each case and stained with mouse anti-PLK1 antibody (Zymed 37-7100) or Rabbit polyclonal anti-FOXM1 antibody (SC-500, Santa Cruz, FoxM1 K19 antibody) as previously described [15]. The slides were scored jointly by JC, MD and SH using a Nikon Eclipse 50 Microscope (*blinded*). The slides were also scanned using a MIRAX 3Dhistotech system for reference and archive purposes. The scores were calculated by subjectively assessing nuclear intensity on a score of zero to three. The percentage of cells showing nuclear staining was documented to the nearest five percent. An overall score was obtained by multiplying the intensity by the percentage.

### RNA isolation and RT–PCR analysis

RNA was extracted, it’s integrity determined and subsequent real-time RT–PCR performed for *PLK1* and *FOXM1* RNA using SYBR Green as described previously [15]. RNA samples from all human tissue specimens were validated using an Agilent 2100 bioanalyser with a RNA 6000 Nano assay lab chip kit. For relative comparison of mRNA levels from tissue specimens, data were further normalised to the level of each gene in a stock standard concentration of RNA isolated from Het1a cells. The cell lines were cultured and lysed as described previously [15].

RT-PCR primers were designed using the internet programs Primer 3 (http://frodo-wi.mit.edu/primer3/input.htm) and NCBI primer blast (http://www.ncbi.nlm.nih.gov/tools/primer-blast/). All primers were designed to cross an intron-extron junction sequence to minimize amplifying genomic DNA contamination. Ribosomal 18S RNA, *β*-*actin*, and *GAPDH* were assessed as housekeeping genes for the normalization of the clinical biopsy samples and samples were ultimately normalized to 18S RNA.

### Statistics

SPSS 16.0 (IBM^®^, New York, USA) was used for statistical analysis. The paired T test was used to compare continuous variables. Chi-Square, Mann–Whitney U (nominal variables with two values) and Kruskal–Wallis (nominal variables with multiple values) were used to compare discrete variables. Kaplan–Meier analysis was used to calculate survival curves by univariate and multivariate analysis respectively. Significance was accepted to be present with a p value of less than 0.05.

## Results

### FOXM1 protein expression

Samples were obtained from 79 patients with gastric adenocarcinomas in a large general hospital with an established cancer surgical service (Table 1). 47 patients had paired normal gastric tissue obtained >5 cm from the edge of the tumour. Tissue microarrays (TMAs) consisting of biopsies of clinical endoscopic and surgical resection specimens were constructed. The expression of FOXM1 was then probed using the FOXM1 K19 antibody.

**Table 1:** Demographics, clinical staging, treatments and FOXM1 protein expression in gastric tissue

	Normal gastric tissue	Gastric adenocarcinoma
Number of cases	47	79
Male	30 (64 %)	46 (58 %) (p = 0.87)
Age	69 ± 9	70 ± 11 (p = 0.94)
Pathological differentiation		
Poor		40 (51)
Moderate		28 (35)
Well		9 (11)
Missing		2 (3)
Depth of invasion		
T1		8 (10)
T2		22 (28)
T3		42 (53)
T4		5 (6)
Missing		1 (1)
Metastatic disease		
Local (N)		53 (67)
Distant (M)		4 (5)
AJCC 2010 stage		
1		15 (19)
2		22 (28)
3		31 (39)
4		9 (11)
Missing		1 (1)
Treatment		
Surgery		65 (82)
Surgery and chemotherapy		5 (6)
Chemotherapy		6 (8)
Palliative care		2 (3)
Missing		1 (1)
FOXM1 mRNA expression		
Positive expression	26 (55)	56 (71) (p = 0.001)
High positive expression	1 (2)	23 (29) (p = 0.001)

The number of cases is illustrated, percentages are in brackets. The number of patients with missing data is indicated. Positive expression was taken as an immunohistochemistry score of > 90 and High positive expression was taken as an immunohistochemistry score > 150. P values calculated by Chi^2^ test are indicated

**Fig. 1:**
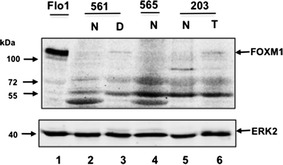
FOXM1 protein expression in gastric tissue. Western blots of FOXM1 protein expression in the indicated tissue type are shown. Molecular weights are in kDa. Results of normal gastric mucosa (N) and Tumour (T) or high grade dysplasia (D) in specimens 571,565,187 from three gastrectomies are shown. Flo1 cell lysate is shown in the *left* lane. ERK2 was used as a loading control

**Fig. 2:**
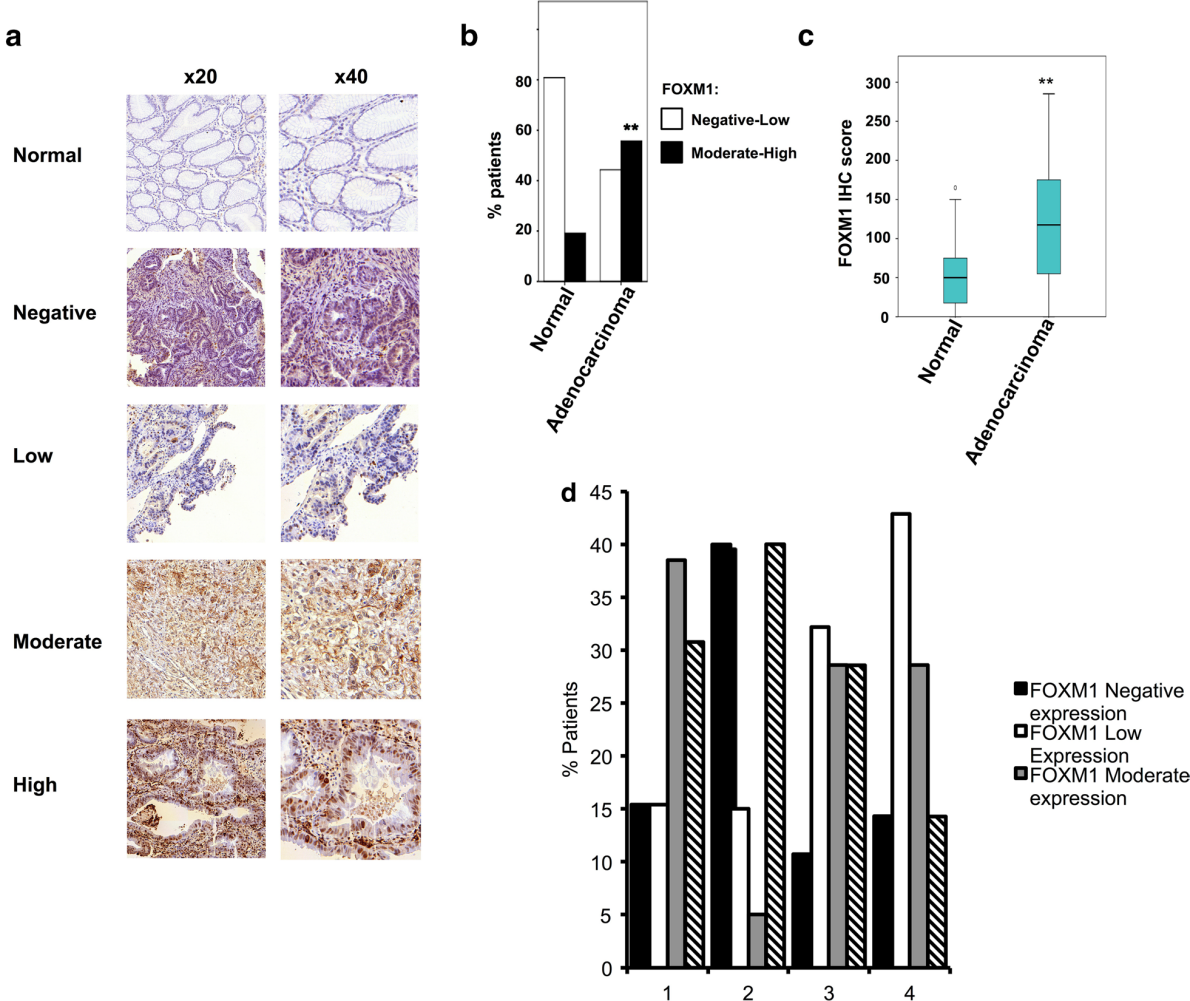
FOXM1 protein expression in gastric tissue assessed by immunohistochemistry. **a** Tissue microarrays stained by FOXM1 antibody showing normal epithelium, negative, low, moderate and high staining of gastric adenocarcinoma at ×20 and ×40. **b** The proportion of patients in each category with negative-low FOXM1 staining (*white bar*) and moderate-high FOXM1 staining (*black bar*) is shown. **c** Boxplot of FOXM1 protein expression in gastric tissue. Median values of FOXM1 expression are indicated for each tissue type (indicated by *horizontal bar*). **P value <0.01. **d** The histogram demonstrates FOXM1 expression (grouped as negative, low, moderate or high) in relation to AJCC stage as assessed by immunohistochemistry

First, to confirm the validity of the FOXM1 antibody and assess protein expression in tumour samples we performed western blot analysis of FOXM1 expression in corresponding tissue types from 3 types of gastrectomy specimens: normal tissue (N), high grade dysplasia (D) and tumour (T). This is suggestive of enhanced levels of FOXM1 protein in both dysplastic tissue and gastric adenocarcinomas (Fig. 1, lanes 3 and 6). Next we analysed FOXM1 protein expression using the TMAs. FOXM1 was shown to cause predominant nuclear staining in gastric adenocarcinomas, although some cytoplasmic staining was seen (Fig. 2a). Staining of varying degrees was seen across the cores. For the negative controls sections we have pre-incubated the antibody with its peptide and there was no staining, hence we are confident of its specificity. As it is a commercially available antibody, we use a standardised positive control and hence confident of the sensitivity. Gastric adenocarcinoma samples had a higher IHC score than paired normal tissues (Fig. 2b) (Paired T test p = 0.001) IHC. Moderate to high FOXM1 expression was significantly associated with gastric adenocarcinoma compared to non-cancer tissue (Mann–Whitney U p = 0.001) (Fig. 2c). FOXM1 positive expression was seen in up to 55 % of the paired normal gastric tissues but of these 65 % were low positive expression. There were no significant differences between FOXM1 expression and tumour depth, nodal metastasis, distant metastasis or AJCC stage (Fig. 2d). FOXM1 expression did not have a correlation between survival or recurrence in gastric adenocarcinomas (data not shown).

### *PLK1* and *FOXM1* mRNA expression in Gastric Adenocarcinoma

Having established up-regulation of FOXM1 protein expression in gastric cancers, we next wanted to test whether we could detect co-upregulation of PLK1. However we were unable to find a suitable antibody for IHC so instead we turned to RT-PCR analysis. We quantified *FOXM1* and *PLK1* mRNA expression using real-time RT-PCR and normalised mRNA levels to 18S ribosomal RNA. Samples from 22 patients were included in the study with patients being divided into tissue groups. The groups included tissue from macroscopically normal gastric epithelium from healthy controls (n = 13) and gastric adenocarcinoma patients (n = 8). The clinical details are indicated in Table 2.

**Table 2:** Demographics, clinical staging, treatments, *PLK1* mRNA and *FOXM1* mRNA expression of the patients in each tissue group

	Gastric normal	Gastric adenocarcinoma
Number of cases	13	8 (1HGD)
Male	4 (31)	7 (88) (p = 0.02)
Age	72 ± 3	70 ± 4 (p = 0.45)
Smoking		
Never	1 (8)	1 (11) (p = 0.45)
Ex-smoker	1 (8)	2 (22) (p = 0.98)
Current	5 (38)	6 (67) (p = 0.09)
Missing	6 (46)	0
Pathological differentiation		
Poor		6 (75)
Moderate		0
Well		0
Missing		2 (25)
Depth of invasion		
T1		1 (13)
T2		2 (25)
T3		3 (50)
T4		0
Missing		1 (13)
Metastatic disease		
Local (N)		3 (37.5)
Distant (M)		0 (0)
AJCC 2010 Stage		
1		2 (25)
2		3 (36)
3		2 (25)
4		0
Missing		1 (13)
Treatment		
Surgery		6 (75)
Surgery and chemotherapy		1 (13)
Chemotherapy		0
Chemo-radiotherapy		0
EMR		0
Palliative care		1 (13)
Missing		0
PLK1 mRNA expression		
mRNA level ×10	2.1	18.8
Positive expression	2 (15)	5 (63) (p = 0.056)
FOXM1 mRNA expression		
mRNA level ×10	1.2	9.2
Positive expression	0 (0)	6 (75) (p = 0.001)

The number of cases is shown in each category, percentages are in brackets. mRNA levels are mean expression levels and standard deviation relative to 18S ribosomal RNA and standards derived from HET1a cell lines. The number of patients with clinical data missing is indicated. Positive expression is defined as an expression level equal or higher to the mean of the normal sample plus two standard deviations about the mean for the indicated gene. P values calculated by Chi^2^ test are indicated

*HGD* high grade dysplasia, *EMR* endoscopic mucosal resection

**Fig. 3:**
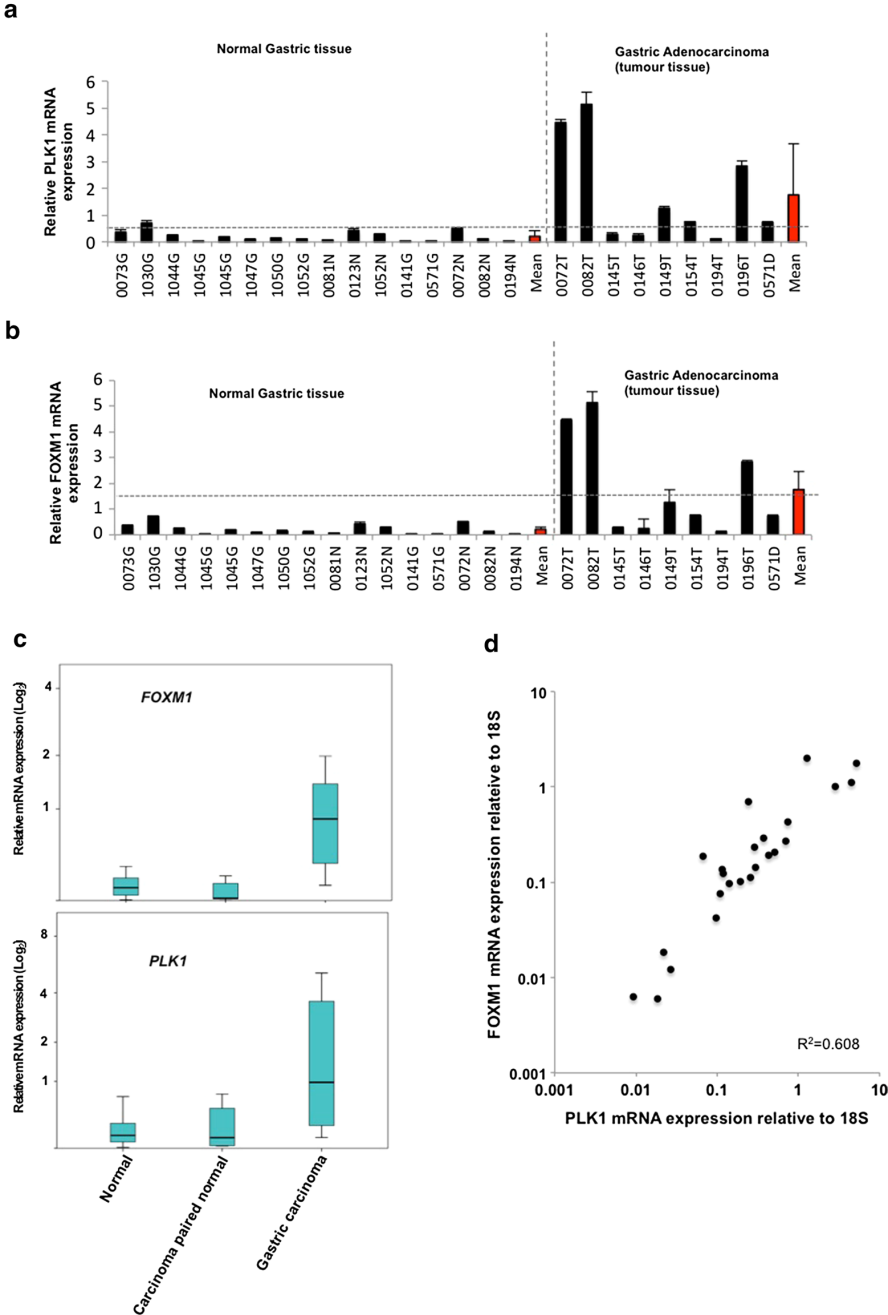
*PLK1* and *FOXM1* expression in normal gastric tissue and gastric adenocarincoma. **a** Histograms of the mRNA levels of *PLK1* and *FOXM1* relative to 18S mRNA in gastric tissue. Data was standardized relative to the gene expression in stock mRNA samples prepared from the Het1a cell line. The average relative mRNA level and standard deviations derived from two readings from one sample are shown. The individual tissue specimens are numbers and samples are grouped by tissue sub-types. The mean gene expression in each category is shown in *red*. Positive expression (calculated as two standard deviations above the mean of the value in normal samples) is illustrated by the *horizontal dotted line*. **b** Box plots of *PLK1* and *FOXM1* mRNA expression. The box plot demonstrates the interquartile range. Median expression values are indicated by the *horizontal lines*. **c** Scatterplot indicating the expression of *FOXM1* relative to *PLK1* mRNA expression. R^2^ value is indicated

The expression levels of both *PLK1* and *FOXM1* mRNA are higher in gastric adenocarcinoma tissue compared to gastric tissue from healthy controls (Fig. 3a, b). Median expression levels are increased ninefold for both *PLK1* (paired T test p = 0.007) and *FOXM1* (paired T test p = 0.001) (Fig. 3c). 5/8 (63 %) tumour samples demonstrated high levels of *PLK1* and *FOXM1*. Importantly there was a strong correlation between *FOXM1* levels with *PLK1* expression levels across all the samples tested (R^2^ = 0.608; Fig. 3d) suggesting a direct co-regulatory relationship. No normal samples expressed high levels of *PLK1* and *FOXM1*. Given the small sample size it is not appropriate to analyse mRNA expression levels for clinical correlations.

## Discussion

In this study we have demonstrated that FOXM1 protein, and *PLK1* and *FOXM1* mRNA levels are all markers of gastric adenocarcinoma when compared to normal gastric tissue. Importantly, we provide evidence for co-upregulation of FOXM1 and PLK1 expression in these cancers as predicted from their co-regulatory association. Previous groups have demonstrated FOXM1 over-expression in cancer [15, 25, 26]. FOXM1 has previously been shown to be overexpressed in 88 % of gastric adenocarcinomas in a cohort of Chinese patients [26]. PLK1 has separately been reported to be overexpressed in gastric cancer patients [22, 27]. Our results are consistent with this and confirm the overexpression of both FOXM1 protein and mRNA in gastric cancer. We did not find any associations with clinical characteristics or prognosis within our immunohistochemistry analysis. Li et al. [25] demonstrated a highly significant association between immunohistochemical expression of FOXM1 and prognosis. Both studies had similar number of therapeutic characteristics. The reason for the differences in prognostic association is unclear. It is possible that there was a difference in the numbers of proximal and distal gastric cancers between the two groups or other factors between our British population that differ from the American population in their study. It can be surmised there was a general consistency between both our protein and RNA results and these overexpression seen by other groups in gastric cancer.

It is perhaps of interest to assess whether PLK1 or FOXM1 have utility aiding with prognostic assessment and identifying populations that might respond to targeted therapies. The data from our Real time RT-PCR analysis of *PLK1* and *FOXM1* in cancer samples did not show a statistically significant survival benefit in either the surgical or non-surgical patient group. This may be as a result of the heterogenicity of the small gastric cancer population in the samples we collected. Clinically homogenous populations are more useful for assessing survival benefit. American and UK groups have previously used microarray data from surgical patients with oesophageal cancer to demonstrate two and four gene signatures that have prognostic value [23, 28]. PLK1 was found to be a potential prognostic marker by one of these studies although this was not further validated [23]. Validation of microarray findings is important because there can be poor correlations between qRT-PCR and normalized microarray data for up to 16 % of genes [29].

PLK1 inhibitors are in active development as oncology treatments [30]. It is possible that PLK1 inhibition will be of clinical utility in treating gastric cancer patients. Initial phase II trials including those utilising BI 2536 have been disappointing with no patients with advanced disease having a complete or partial response [31]. Drug limiting effects including neutropenia, thrombocytopenia and anaemia were observed frequently. It is therefore unsurprising that the major limiting side effects relate to impairment of rapidly proliferating tissues given PLK1’s major role in regulating the cell cycle. Whilst patients did not gain a significant clinical response to BI2536 treatment it is worth noting that some tumour types did enter a period of stable non-progressive disease, particularly ovarian cancer patients [31]. A recent phase I clinical trial which used another PLK1 inhibitor GSK461364, found that stable disease was achieved for greater that 4 months in 6/40 patients [32]. Interestingly all of the responders to treatment had significantly higher PLK1 and KI67 levels when assessed by immunohistochemistry. This may suggest PLK1 inhibition may be of most use in the subset of patients with overexpression of FOXM1 and it’s target genes.

## Conclusion

It is clear that both PLK1 and FOXM1 are tightly regulated to prevent inappropriate proliferation, and both have widespread interactions throughout the cell cycle. Carcinogenesis is a dynamic process with changes in the genome, transcriptome, intra/extracellular signalling throughout the tumour, as well as alterations to the surrounding vascular and stromal tissue. Whilst our results suggest that both PLK1 and FOXM1 are implicated in carcinogenesis in gastric adenocarcinoma, blockade of one regulatory pathway is unlikely to halt cancer progression and induce widespread apoptosis in vivo. Increasingly more recent cancer therapies often involve blockade of multiple pathways using a combination of therapeutic agents and it may be that targeting FOXM1 pathways will prove a useful treatment adjunct.

Work is already being undertaken in our laboratory to identify new FOXM1 target genes using ChIP-seq technologies. Future work will focus on using gene expression array data to assess alternative FOXM1 targets and novel FOXM1 interactions in gastric cancer. FOXM1’s multiple potential roles in carcinogenesis suggest that the development of chemotherapy that targets the FOXM1 regulatory network may have clinical utility.
